# Observations on the Visual Fields and Early Fundus Changes in Retinitis Pigmentosa

**Published:** 1895-08

**Authors:** Alex. W. Stirling

**Affiliations:** Atlanta, Ga.


### OBSERVATIONS ON THE VISUAL FIELDS AND EARLY FUNDUS CHANGES IN RETINITIS PIGMENTOSA.

BY ALEX. W. STIRLING, M.D.; Honors—M.B. and C.M. (Edin.), D.P.H. (Lond.),

ATLANTA, GA.

In this short paper I do not propose to discuss in detail the subject of retinitis pigmentosa. That has been done satisfactorily in all the good text-books on ophthalmology. My desire is rather to lay before the reader one or two points which I happen personally to have observed, and which are not so described in the text-books. At the same time it may be well to draw in a few words a rough outline of the general features of the disease.

Retinitis pigmentosa is probably one of the commoner of the rare diseases of the eye, and is said to be found more often in males than females. It is well defined, and may generally be diagnosed before an ophthalmoscopic examination has been made, even though its pathology is confined almost entirely to the fundus, from the history of a slowly progressive inability to see in diminished light, affecting therefore, both eyes, the patient, for instance, seeing "fairly well" by daylight, but being unable to count fingers by twilight; and of a gradual contraction of the field of vision, till in the late stages, while possibly still able to read ordinary print, he may yet be unable to move about in safety by himself, because he sees no more than if he were looking through a narrow tube. The defect is generally first recognized about puberty or early adult life, and sight is apt to be entirely gone by late middle life.

These are the main subjective symptoms, and they are not unfrequently present in more than one member of a family, and are said to be often associated with other defects which are congenital, such as deficiency in mental power, deaf-mutism, cleft palate, and so on, all of which are considered to be specially frequent among children whose parents are blood relations.

Objectively, one finds, and all the books describe, certain distinctive features in the fundus, the most noticeable of which is an arrangement of the pigment in the anterior layers of the retina, occupying a more or less peripheral zone, and made up of a delicate reticulum, which it is customary to compare to the Haversian bone corpuscular system, the pigment also tending to follow the course of vessels, especially of veins, this extra deposit being associated with a loss of pigment in the layer of hexagonal cells on the posterior retinal surface, the choroid coming thus to view in an unusual degree.

Another striking feature is the change in the retinal arteries and veins, whose lumen, in confirmed cases, is much diminished in size; along with the pale, degenerated appearance of the disc, which more resembles wax than the white of the ordinary forms of optic atrophy; while sometimes associated with these changes is found posterier polar cataract.

There are rare cases which are exceptions in one or more of the above particulars: for instance, central vision has been found to be more and earlier affected than peripheral; the pigment has been absent from the periphery and present in its distinctive form at and around the macula; a disease, in every other way resembling this, is sometimes observed, but without the pigment changes. One such I have seen. Also, it is well to bear in mind that though both eyes are usually affected, the disease may be less advanced in one, or confined entirely to it, so that the visual symptoms may be delayed or greatly minimized. For example, I have seen a case in a girl who complained of no night blindness, because the retina was nearly healthy in one eye, though a defective light sense was quite evident with Foerster's photometer. Further anomalies have been described by Germain and Dianoux.*

The only disease likely to be mistaken for this is syphilitic retino-choroiditis, but it is only rarely that the diagnosis is difficult.

Retinitis pigmentosa in itself presents no good reason for excision of the eye; therefore it is comparatively rarely that it comes up for microscopical examination. Its pathology is seen to be as little of an inflammation as the clinical picture would lead one to expect. It is rearly a sclerosis and degeneration, beginning either in the retina itself, or in the choroid. Wagenmann † describes

* Ann. d'Ocul., October, 1893, quoted in *New York Medical Journal* by Bull, March 2, '95.
† Graefe's Archiv. XXXVII, i. p. 230.

the choroidal vessels as affected by endarteritis, with hypertrophy of their coats and obliteration of their lumen; and, for reasons to be presently mentioned, it appears to me probable that this is the tunic in which the pathological process has its origin. In the retina the rods and cones, nervous layers, vessels, etc., are undergoing destruction, while their place is being occupied by a fibrous tissue. The pigment layer is also disappearing, while the coloring matter is wandering into the anterior layers of the retina, where possibly it increases in amount by proliferation.

The causes of this affection cannot be said to have been definitely ascertained. That there is some family predisposition at the bottom of it, is fairly well established, for it occurs with exceptional frequency in those nearly related. That this taint is syphilitic is improbable, but it is asserted that its victims are frequently the offspring of marriage between blood relations.

Concerning its treatment I am not encouraged to say anything whatever, beyond mentioning the fact that the constant current, advocated by Mr. Marcus Gunn, has been of temporary benefit in some cases.

Of the points to which I desire specially to refer the field of vision is the first. We know that the normal eye, when steadily fixing a point in front of it, receives impressions also from all objects which lie within a certain area around that point. That area, called the field of vision, for each eye separately extends outwards for a white object more than 90°, upwards between 50° and 60°, downwards between 60° and 70°, and inwards between 45° and 55°; and that, for colors, at any rate as ordinarily used, the field is somewhat smaller, and for blue, red, and green—those generally employed in testing, the diminution proceeds by fairly equal steps in the above order till for green the field has come down to about 75° outwards, 33° upwards, 47° downwards, and 27° inwards. It has, however, been shown that the areas apparently non-sensitive to colors are not really entirely so; for, with the proper brilliancy of hue in an abundant light, the field for them is as great as that for white. The extent of the field is also affected by the contour of the face, a prominent nose and overhanging brows interfering with its size inwards and above. Inward the fields of the two eyes overlap.

Now, in certain diseases, such as glaucoma and optic atrophy, the size of the field is reduced, and in none more markedly than in retinitis pigmentosa. It is this contraction of the seeing area of the retina which, though it is almost always accompanied by a certain falling off in central vision, may yet, while the patient is still able to read with comfort, cut him off from all independent locomotion, for the safe performance of which the peripheral parts of the retina are very necessary.

The order of contraction is usually stated to be that it begins at the periphery, and gradually passes inward till the macula itself is involved, and even central vision disappears, leaving the eye completely blind. It has been occasionally observed that a small peripheral zone, or a patch on the temporal side, may continue longest sensitive, but authorities generally describe the contraction as affecting the whole periphery, and gradually extending towards the center.

With the object of testing the accuracy of this statement I have carefully examined the field in some of the cases which have come under my observation, with the result that I am led to believe it is the common thing for the field to contract in a much less regular form than that generally described. I have at hand only six charts, from the two eyes of three patients, but these are sufficient for illustration.

CASE 1. A woman, aged forty-six, who was handed over to my care when house surgeon in the Royal Westminster Ophthalmic Hospital, London. Besides the usual features of the disease she had a few small vacuoles and a posterior polar cataract in each lens. Her right field of vision is so contracted towards the center that it reaches only to 5° all around the macula, except on the temporal side where it extends as far as 8°. But beyond this, a little down and to the temporal side, there remains a small oval island of sentient retina, the breadth of which fills in that position the space between the thirtieth and fortieth circles, while still farther out, and in the same direction and of the same shape, is another such island about four times the size of the former, and whose width occupies a space between the sixtieth and eightieth circles.

The left eye shows a field contracted concentrically to the same extent as the right, withon the temporal side, a seeing area of a roughly oval form, whose length from above downwards is about double its breadth, which extends from 55° to 80°. The condition of the lenses could have had nothing to do with the form of the fields.

Case 2. A man, aged twenty-nine. The right field extends on the nasal side to 18°, upwards to 15°, downwards to about 20° at one point, and on the temporal side to 22°, the lines joining these points forming, however, a very irregular figure. Downward and to the temporal side is a roughly oval island of seeing retina about double as long as it is broad, the breadth filling a space between the sixtieth and eightieth circles. The left field resembles pretty closely the right, except that the island, also on the temporal side, is at a higher level, reaches at its widest part between 55° and 90°, and is twice as long as the other.

Case 3. A woman, aged twenty-five, who came last summer to my clinic in the Post-Graduate Hospital, New York. In her right eye she still had a fairly good field, reaching to 40° on the nasal side, upwards and downwards, and to 55° on the temporal side. This chart presents no islands, perhaps because the field is still so large. I am not responsible, however, for its correctness, as it was taken in my absence by one of the students. That of the left eye, for which I am responsible, shows a field inwards of 45°, upwards of 40°, downwards of 50°, and outwards of 40° to 45°, and also a *non-seeing zone* of 5° to 10° in width roughly between the thirtieth and fortieth circles towards the nasal side, and between the twentieth and thirtieth circles on the temporal, except upwards and outwards, where it runs into the main body of the insentient area. (This case will come up again along with Case 5.)

It will be noted that here, as in glaucoma and in optic atrophy, the temporal side of the retina is more prone to lose its functions than the nasal.

Case 4 introduces the second point, which appears to me worthy of special consideration.

The mother of Case 4 exhibited the ordinary symptoms of retinitis pigmentosa of middle age, and said that both her mother and her mother's mother had lost their vision in a way precisely similar to her own. Her fundus presented the usual appearances.

Among other things, the pigment was deposited in the well-known stellate form.

With the idea of seeing a case before symptoms had set in, I obtained permission to examine the eyes of her daughter, Case 4, a girl of twelve years of age. It was not convenient to take the fields with the perimeter, but taken roughly, they were full; no history of any night blindness could be elicited; her vision, so far as I was able to tell under the circumstances, was perfect. She had no complaint whatever, and was possessed, apparently, of absolutely healthy eyes, although I am of opinion that a thorough examination would have revealed some defect too slight for her own observation. With the ophthalmoscope a striking picture was revealed. The disc and vessels were normal, but the general aspect of the fundus was peculiar, for it appeared as if sprinkled thickly over with black pepper, the grains of which were, however, somewhat more numerous toward the periphery, where also there were one or two deposits of the typical bone corpuscular shape. The black points were scattered throughout the thickness of the retina, and were too thickly strewn to permit of a good view of what lay behind them.

CASE 5 AND CASE 3 CONTINUED. These are sisters. Here there is no family history of eye diseases; the parents were not related, and there is no congenital abnormality in any member of the family. The eyes began to trouble Case 3 when she was about twenty years old (she is now twenty-five), when they became painful and "burned." The usual visual defects of retinitis pigmentosa have recently been present, but to a limited extent, and these, she thinks, are not progressing. Vision in each eye was about $\frac{6}{12}$. So far as my notes carry me, and from a letter just received from the patient, I gather that the burning pain was, and still is, her chief complaint.

Besides the contracted condition of the fields already described, and a slight pallor of the discs, I found in both fundi, but more marked in the left, a few typical masses of pigment arranged along the vessels, and in both a black punctate condition of the retina resembling that seen in Case 4. This patient has three brothers, aged respectively twenty-seven, twenty-four, and ten years, and one sister aged nineteen years. To quote her own words, "none of them complain in any way about their eyes, or not seeing well." Although I hope later to have a report on the brothers' eyes, so far I am able to speak concerning the sister only. She (Case 5) came, at my request, along with her elder sister, on her next visit to the clinic. I regret that I have no notes concerning any perimetric examination in her case; but, as already said, even with leading questions, no eye symptoms could be elicited. Vision in each eye was $\frac{6}{5}$. The ophthalmoscope showed that she, too, had the peppery condition of the fundus, but in her it was most marked at the nasal side of the disc in each eye.

The appearance described in the eyes of these last cases strikes me as being of considerable interest, chiefly for two reasons: First, it is not improbable that this may be a usual step towards the formation of the typical retinal picture. At all events, although I have examined with the ophthalmoscope a good many thousands of eyes, I have never seen any other condition at all resembling this. I am acquainted with the very various aspects of the normal fundus, and know that congenital patches of pigment are sometimes found in the retina, as well as that the shape of the deposits in retinitis pigmentosa is not always quite according to classical descriptions; but I had never before seen, nor heard of, such an appearance as this, either in diseased or healthy eyes, while it is remarkable that I should have seen it in two healthy young persons in whose family retinitis pigmentosa existed, and in one to whose eyes attention had been drawn chiefly by symptoms other than those of the not far advanced retinitis pigmentosa, which was present.

My idea is that this is the early stage of the disease, when the pigment is being set loose, and is travelling forwards into the anterior layers of the retina, before it has left the choroid bare and has settled down in the periphery among the nerve tissue, from which it receives its typical form, and before the degenerative process has destroyed the essential visual elements. In support of this are the facts that in Case 4 we have a very marked family history of hereditary retinitis pigmentosa, and that in her the pigment had begun to take on the typical form, for there were already one or two stellate deposits at the periphery; that in the mother's eyes the deposit was typical; and also that the position of the pigment must almost certainly be temporary, for it is surely the rarest thing, if it is not altogether unknown, to have the pigment so equally deposited over the fundus in a case which is advanced. Then Case 3 and Case 5 are sisters, and the elder woman, with slight though distinct symptoms, had the peppery fundus along with typical pigment masses, a more advanced condition than that of the younger, who, with no symptoms, had the peppery fundus only.

The second reason why I find the appearance of special interest, is because it may be of some service in helping to settle the question of the position in which the pathological process has its origin. Had there been pathological changes of any extent in the substance of the retina itself, even if invisible with the ophthalmoscope, they would certainly have given rise to symptoms. The only apparent change being the displacement of the pigment, it follows that the diseased process in all probability begins either in the layer of hexagonal pigment cells itself, or more probably in one of the adjacent layers of the choroid, the vessels of which have in retinitis pigmentosa, as a matter of fact, been found to be subject to endarteritis with secondary obliteration. Then, if the hexagonal pigment layer be the position of primary attack, how are we to account for those rare cases where the pigmentary changes are absent?

It is constantly repeated that the victims of this disease are frequently the children of parents related by blood. Had it not been that such observers as Hutchinson and Leber have found this to be so in twenty-five per cent. of cases, I should have thought it an exaggerated statement; for, while I believe I have inquired concerning this point in every case I have seen, I cannot remember ever to have received a positively affirmative statement, though it must be admitted that patients are sometimes themselves a little hazy on the subject. MacNamara also has pointed out that the disease is frequent among Hindus, where consanguinity in marriage is forbidden.

The main object in all medical research being the relief of suffering, we should endeavor to turn even the smallest contribution to our knowledge to some practical account. The only suggestion which may here be to the point, is that it would be well that the younger members of any family in which retinitis pigmentosa makes its appearance should be very carefully examined for any symptoms of it, in order that treatment may be employed at the earliest possible moment. For, it does not follow that, because up to the present time successes have been rare in endeavoring to stop the relentless course of this disease, science may not at some future time, let us hope before long, discover some more hopeful method ; but whatever that may be, it will have to be applied while the ocular tissues are still comparatively healthy, before a fibrous has replaced the normal nervous tissue.

